# The role of models in translating within-host dynamics to parasite evolution

**DOI:** 10.1017/S0031182015000815

**Published:** 2015-09-24

**Authors:** MEGAN A. GREISCHAR, SARAH E. REECE, NICOLE MIDEO

**Affiliations:** 1 Department of Ecology and Evolutionary Biology, University of Toronto, Toronto, ON M5S 3B2, Canada; 2 Institutes of Evolutionary Biology, and Immunology and Infection Research, University of Edinburgh, Edinburgh EH9 3FL, Scotland, UK

**Keywords:** Within-host ecology, fitness, transmission, malaria

## Abstract

Mathematical modelling provides an effective way to challenge conventional wisdom about parasite evolution and investigate why parasites ‘do what they do’ within the host. Models can reveal when intuition cannot explain observed patterns, when more complicated biology must be considered, and when experimental and statistical methods are likely to mislead. We describe how models of within-host infection dynamics can refine experimental design, and focus on the case study of malaria to highlight how integration between models and data can guide understanding of parasite fitness in three areas: (1) the adaptive significance of chronic infections; (2) the potential for tradeoffs between virulence and transmission; and (3) the implications of within-vector dynamics. We emphasize that models are often useful when they highlight unexpected patterns in parasite evolution, revealing instead why intuition yields the wrong answer and what combination of theory and data are needed to advance understanding.

## INTRODUCTION

Within-host infection dynamics are thought to matter critically for evolutionary outcomes of interest, for example, whether drug resistance will emerge in a parasite population (e.g. Day *et al.* 2015), or whether parasites will evolve higher virulence (reviewed in Chao *et al.* 2000). Intuition is a tempting tool for discerning the selective benefit of parasite traits, and for predicting how within-host processes will scale up to influence patterns in parasite evolution. But intuition is frequently misguided, and theoretical models offer great utility at every stage: formalizing intuition so that it can be tested, identifying what experiments and statistical methods are needed to evaluate those expectations and synthesizing experimental findings into a coherent narrative that can be applied broadly. Here we review recent examples of how models have been used with varying degrees of success to link parasite traits to within-host dynamics and parasite evolution, suggesting best practices for moving forward and identifying key open questions.

## REFINING STUDY DESIGN FOR WITHIN-HOST ECOLOGY

Predicting parasite evolution often requires understanding within-host processes that are difficult or impossible to observe directly, and the optimal study design for illuminating key aspects of within-host ecology is by no means intuitive. Many factors change simultaneously over the course of infection, including parasite numbers and the availability of host resources, obscuring the effect of experimental perturbations and making it difficult to distinguish interesting biology from noise. Models can greatly extend the utility of experimental work by highlighting the treatments, response variables and statistical approaches most likely to inform understanding. For example, models can help ensure that experiments have enough statistical power to test theoretical expectations by trimming the number of treatments down to those likely to be the most essential, fruitful, or interesting. Models have been used to select the anti-malarial drug regimens most likely to reduce parasite burdens in mice while limiting selection for drug resistant parasites so that a manageable number of treatment protocols could be investigated experimentally (Huijben *et al.* 2013, further examples reviewed in Lessler *et al.* 2015).

**Fig. 1.**
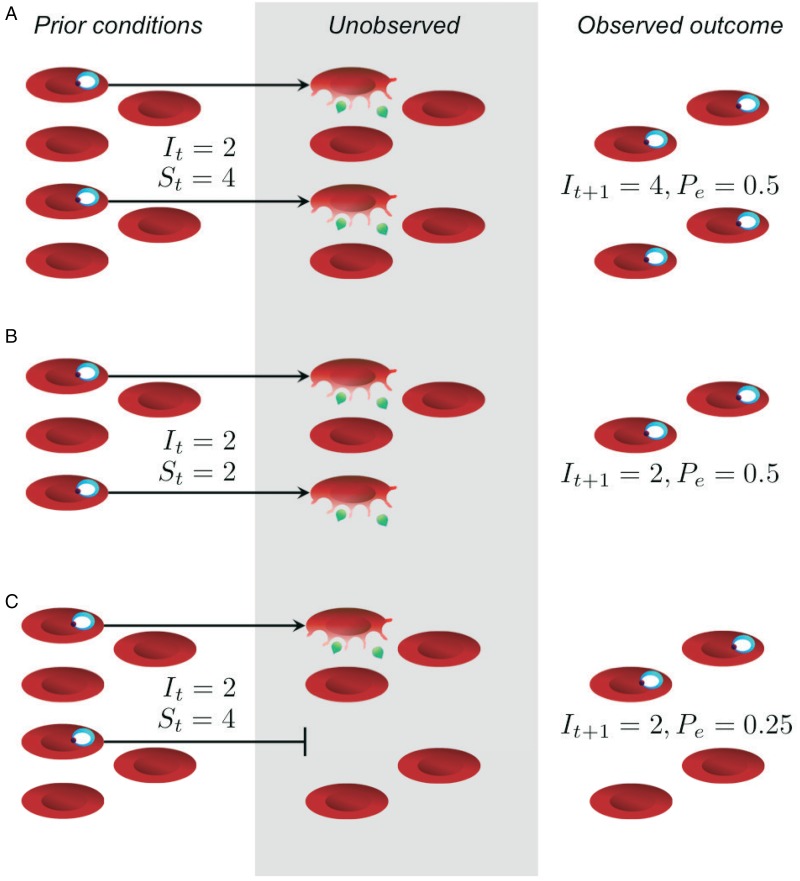
The effective propagation number offers a way to decouple parasite proliferation from resource availability. In (A) and (B), the different outcomes observed at time *t* + 1 can be entirely explained by differences in the starting number of uninfected red blood cells, *S*_*t*_, and infected red blood cells, *I_t_*, with no need to invoke immune clearance; thus, the effective propagation numbers (*P*_*e*_) are the same in both scenarios. In (C), resources were sufficiently abundant, but the outcome was a smaller number of infected red blood cells than expected, reflected in a smaller effective propagation number. Importantly, the greatest fold-change in parasitemia and parasite density occurs in (A), and, without knowledge of red blood cell densities, one might incorrectly infer that immunity is having a greater impact in both (B) and (C). While *P*_*e*_ would typically be estimated by regression (Metcalf *et al.* 2011), we calculate it here assuming that *P*_*e*_ = *I*_*t* + 1_*/*(*I*_*t*_ *S*_*t*_).

Beyond issues of statistical power and experimenter effort, models can be used to identify the appropriate null expectation for comparison, a prerequisite for detecting complicated interactions within the host. In the context of malaria infections, parasites proliferate within red blood cells, so a null hypothesis might be that infection dynamics are driven solely by the availability of host resources, i.e. red blood cells. From experimental infections in mice, detailed data are available for red blood cell numbers and parasite abundance over the course of infection. In the absence of equally detailed data on immune responses, inferring the role of immunity requires specialized statistical methods. Metcalf *et al.* (2011) describe one such approach, which relies on careful accounting of red blood cells and parasites to identify cases when the number of new infected cells generated cannot plausibly be explained by resource availability alone and immunity is likely to be involved (Fig. 1). This work has led to some important inferences by quantifying deviations from the null hypothesis. First, parasite proliferation increases with initial abundance, presumably because innate immune responses are more easily overwhelmed by larger numbers of parasites (Metcalf *et al.* 2011). Second, adaptive immunity plays an important role later in infection, where it appears to be upregulated more quickly in response to larger doses of parasites (see Fig. 3 in Metcalf *et al.* 2011). Extending this approach allows anaemia during infection to be partitioned into losses from parasite exploitation and losses from host responses. The model that best fits the data predicts that hosts reduce red blood cell availability in response to infection by more virulent strains; counterintuitively, anaemia may therefore represent adaptation by the host to reduce parasite replication (Metcalf *et al.* 2012), in line with theory (Cromer *et al.* 2009). These approaches provide a powerful way to distinguish between the potential drivers of infection dynamics – for example, resource limitation *vs* immune clearance or host- *vs* parasite-mediated destruction of red blood cells – by leveraging time series data. Critically, none of these insights about host–parasite interactions would have been apparent from examining parasitemia alone (Fig. 1), underscoring the need for experiments that quantify how parasite numbers and the in-host environment change over the course of infection.

Models can also generate understanding of how within-host dynamics manifest as population level patterns, an area where the null hypothesis is often unclear. For example, intuition might suggest that if there is facilitation between parasite species, whereby infection by one species increases the odds of infection by the other, then they would occur together in hosts more often than is expected by chance. Yet parasite species might also occur more often than expected by chance if some hosts are especially prone to infection, or if there are seasonal changes in exposure risk (Poulin, 2001). In both cases, hosts would tend to be either uninfected or infected with multiple parasite species, making it appear as though parasites benefit from the presence of coinfecting species even when interactions are neutral or mildly negative. Experimentally perturbing coinfections in wild hosts can uncover the chain of causality (or lack thereof), but such field studies are logistically challenging (e.g. Fenton *et al.* 2014), and observational data are often gathered instead (reviewed in Poulin, 2001). Models suggest several challenges in detecting interspecies interactions: for example, even if infection with one parasite species facilitates establishment by another, there may be a maximum number of parasites that can inhabit the same host. Assuming both facilitation and a maximum parasite burden, models have been used to synthesize data on parasite prevalence within the host population (Bottomley *et al.* 2005). These simulations reveal that, as hosts age they may become saturated with parasites and show no evidence of facilitation that might have been apparent in younger hosts, underscoring the need to sample hosts of different ages. Instead of facilitation, parasite species may antagonize one another, but these interactions can be especially challenging to detect when host exposure increases with age, generating a positive association in infection by the two species (Fenton *et al.* 2010). Correlation-based approaches are therefore unlikely to be reliable in the absence of other data on the nature of the interaction between two parasite species (a finding further supported by field experiments, Fenton *et al.* 2014), and more sophisticated approaches may be required (Fenton *et al.* 2010). Thus, a growing number of studies show that using models to synthesize within-host data, where the true answer is known, represents a powerful way to improve study design and statistical methods so that the strength and nature of parasite interactions can be tested.

## INTERROGATING INTUITION

The main goal of many models of within-host infection dynamics is to predict parasite evolution given a set of assumptions about how hosts and parasites interact. Selection is generally expected to increase *R*_0_ (defined as the number of secondary infections, e.g. May and Anderson, 1983), but intuition is rarely sufficient to predict the evolution of parasite traits underlying transmission because their consequences accumulate over the parasite's entire life cycle. We focus on malaria infections, where complex ecology – within the host and the vector – often serves to befuddle intuition regarding the evolutionary consequences of parasite traits. One major challenge is that malaria parasites employ specialized transmission forms (gametocytes) that cannot contribute to within-host proliferation (reviewed in Bousema and Drakeley, 2011). This type of specialization erodes any simple expected relationship between transmission and total parasite abundance within the host and is present in diverse parasites. In addition to the malaria genus *Plasmodium*, all other Apicomplexans utilize special forms for transmission (e.g. *Eimeria* spp., *Babesia* spp., *Toxoplasma* spp., reviewed in Smith *et al.* 2002), as do some trypanosomes (reviewed in MacGregor *et al.* 2012), and certain viruses, bacteria and fungi (reviewed in Anderson and May, 1981). The connection between within-host abundance and between-host spread can be further complicated when transmission investment (allocation to transmission stage production) varies across strains and in-host environments as it does in malaria (e.g. Pollitt *et al.* 2011*b*; Cameron *et al.* 2012, reviewed in Carter *et al.* 2013). Below, we discuss several examples of how intuition about parasite fitness can be overturned by integrating data and models, an approach that serves to illustrate not just when intuition fails, but why.

### Intuition: longer infections enhance parasite fitness

**Fig. 2.**
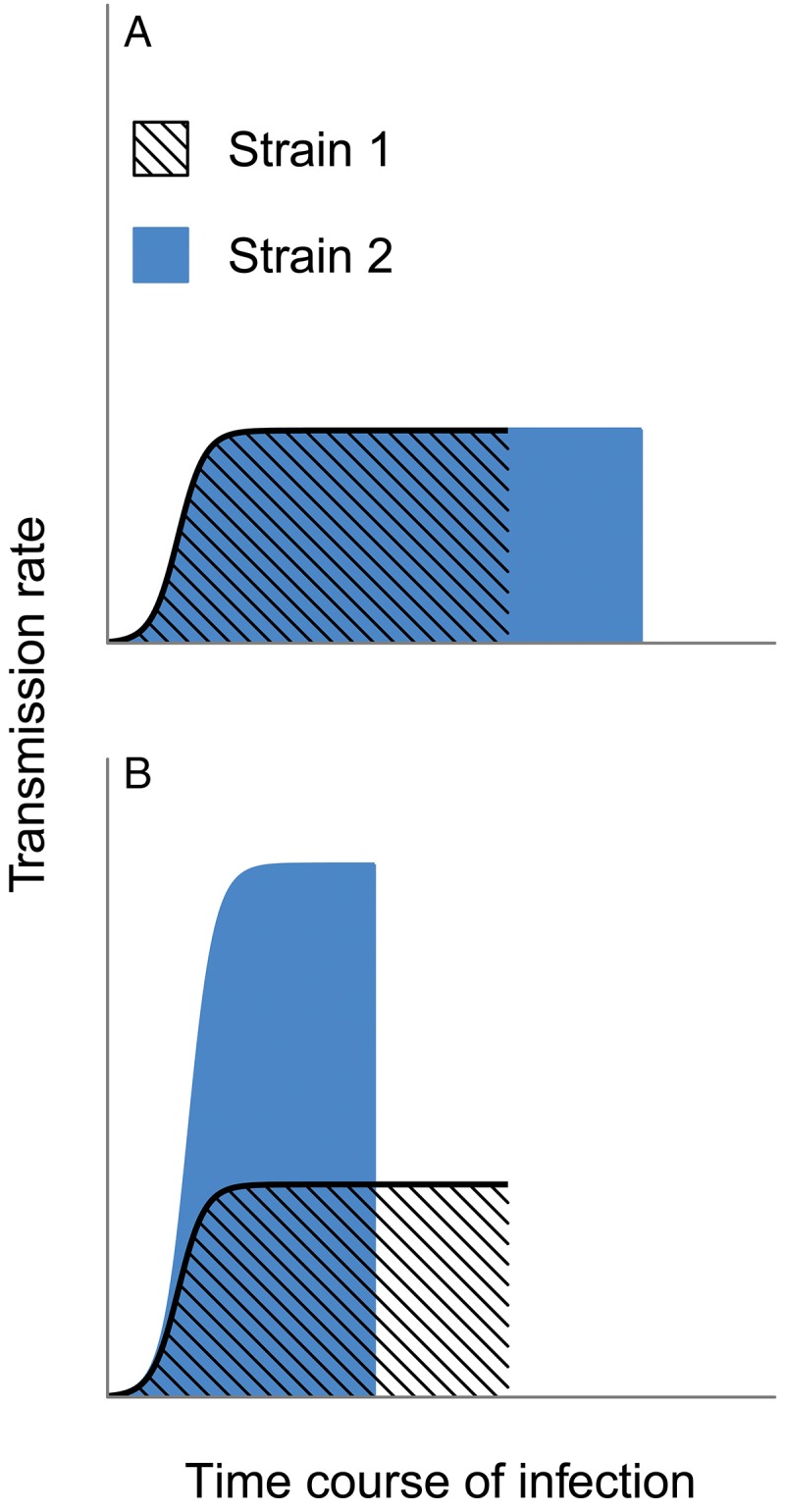
Selection need not maximize the duration of infection. Two hypothetical strains differ in their transmission rates over the course of infection, and selection would be expected to maximize the lifetime transmission success. In panel A, both strains maintain identical transmission rates through time, except that Strain 2 can maintain infection (and transmission) longer and would hence be favoured by selection. In contrast, panel B assumes that shorter infections allow substantially higher rates of transmission. Strain 2 has the greatest cumulative transmission success, despite causing a shorter infection (e.g. by killing the host more quickly), and should thus be favoured over Strain 1.

Malaria infections can persist for hundreds of days (Miller *et al.* 1994) and these chronic infections may be necessary to sustain malaria transmission where mosquitoes are only present for part of the year (reviewed in Bousema and Drakeley, 2011). Many studies assert that a longer period of infectiousness (i.e. of harbouring gametocytes) will increase transmission to mosquitoes (Snounou *et al.* 2000; Recker *et al.* 2011; Eksi *et al.* 2012; Klein *et al.* 2012; Coleman *et al.* 2014; Morahan and Garcia-Bustos, 2014). As a corollary, any trait that increases the period of infectiousness is thus expected to enhance parasite fitness (e.g. schematic shown in Fig. 2A). But data and theory both reveal severe flaws in this logic. Most pressingly, transmission rates are not binary: the presence of gametocytes does not ensure transmission, and data show that over a considerable range, greater numbers of gametocytes improve the odds of infecting mosquitoes (Paul *et al.* 2007; Huijben *et al.* 2010; Bell *et al.* 2012, reviewed in Bousema and Drakeley 2011). Therefore, fitness will not necessarily be enhanced by simply increasing the length of infections, particularly if greater cumulative transmission can be achieved by truncating infection early (e.g. Fig. 2B). Instead, selection will act on the transmission rate integrated over the lifespan of an infection. Given the complexity of within-host dynamics, the idea that a parasite strain could persist longer inside a host with no other alteration to its dynamics and transmission success (Fig. 2A) is likely to be a gross oversimplification and extremely misleading.

Understanding how infection duration and transmission rates are related is further complicated in malaria infections since the traits underlying these processes may be mechanistically linked. For malaria parasites, survival within the host depends in part on antigenic switching (reviewed in Scherf *et al.* 2008). In the human malaria parasite *Plasmodium falciparum*, proliferative forms express proteins encoded by the *var* gene family which over time leads to a protective adaptive immune response (reviewed in Miller *et al.* 2002). Each replicating parasite expresses a single *var* gene (Chen *et al.* 1998), and clonal populations of *P. falciparum* parasites tend to express one *var* gene at a time within the host (Kaestli *et al.* 2004). Recent *in vitro* assays suggest antigenic switching and gametocyte production share some common molecular machinery (Brancucci *et al.* 2014; Coleman *et al.* 2014), raising the question of whether parasites are able to regulate gametocyte production independently from antigenic switching. Models predict that complex antigen switching behaviour could generate chronic infections (e.g. switching order determined by a structured network, Recker *et al.* 2011). Further, models suggest that initially high rates of proliferation could generate a robust immune response against a particular antigen, simultaneously preventing a specific immune response against distinct but structurally-similar antigens; when the initial antibody response wanes, parasites expressing those related antigens will be free to proliferate, prolonging the infection (Klein *et al.* 2014). If gametocyte development functions as an additional ‘antigen’, the switching pattern could profoundly alter transmission potential. For example, in a simple scenario, antigenic switching and gametocyte production could be constrained to proceed at the same rate. In this case, a low rate of antigenic switching/gametocyte production might be strongly favoured early in infection, since theory predicts that rapid initial proliferation greatly enhances future opportunities for gametocyte production (Koella and Antia, 1995) and allows better modulation of the adaptive immune response to permit longer infections (Klein *et al.* 2014). However, later in infection, selection pressures might conflict: in the face of an upregulated adaptive immune response, parasites might do best to restrain gametocyte production so as to proliferate enough to persist within the host (as predicted by Pollitt *et al.* 2011*a*), but a faster rate of antigenic switching might be beneficial to escape the same immune response. More complex scenarios are plausible. Some selective conflicts would be avoided if parasites could regulate antigenic switching independently from gametocyte production. Thus, the mechanistic links between infection duration and transmission potential demand further study. Combining empirical and theoretical approaches could uncover important constraints on malaria parasite evolution that would be difficult to discern from experiments alone.

**Fig. 3.**
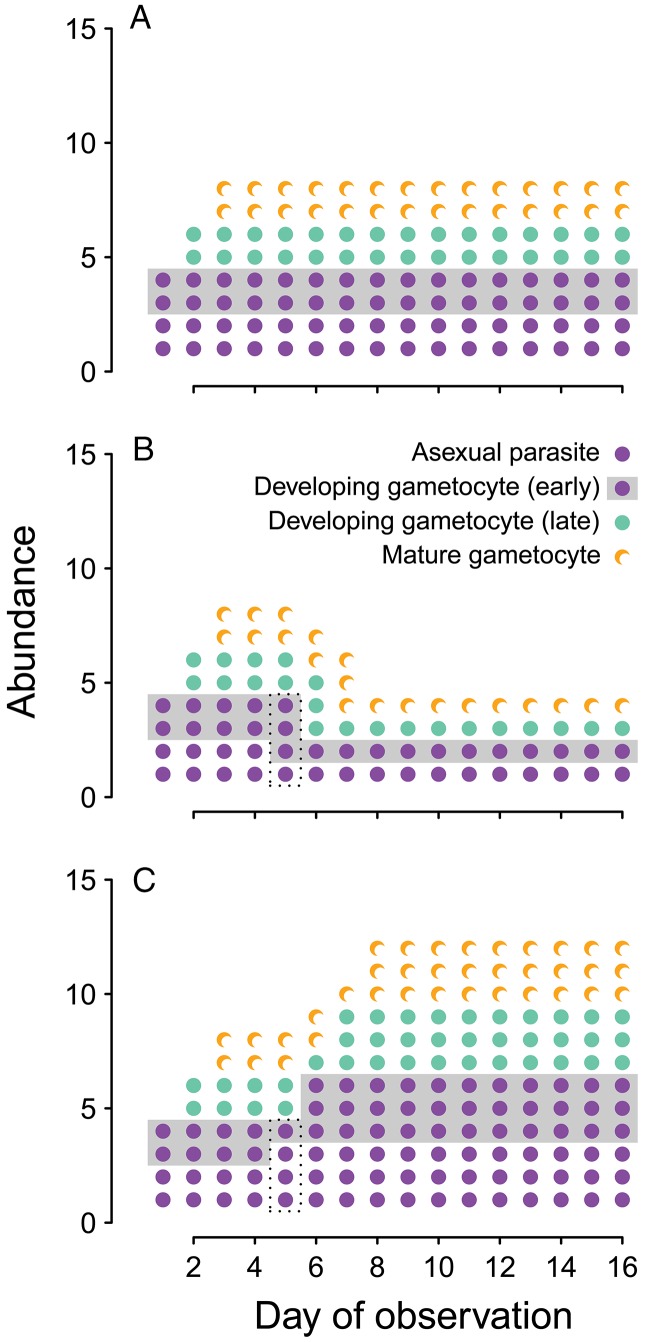
Transmission investment changes within-host dynamics. We assume each life stage (asexual, early gametocyte development, late gametocyte development, gametocyte maturity) lasts only one day (as has been reported for *Plasmodium chabaudi* infections of mice, Landau and Boulard, 1978; Reece *et al.* 2003). For visual clarity, we have deliberately oversimplified the malaria biology: we assume that at first observation infections consist of two asexual parasites and two developing gametocytes, that each asexual produces two progeny, and that each of those progeny can develop as either an asexual parasite or a gametocyte. In (A), investing 50% of progeny into gametocyte production exactly balances the 2-fold replicative capacity of the asexual parasites, leading to constant parasite biomass and gametocyte abundance. In (B), transmission investment is increased on day 4 only, resulting in more developing gametocytes on day 5 (boxed) and a transient increase in mature gametocytes on day 7, followed by a reduction in parasite biomass and gametocyte numbers. Finally, if transmission investment is decreased on day 4 only (C), it leads to fewer developing gametocytes on day 5 (boxed), a transient reduction in mature gametocytes on day 7, and, subsequently, a sustained increase in gametocyte numbers and total parasite biomass.

In the meantime, the fitness consequences of changing transmission investment cannot be adequately assessed by assuming efficient transmission over a threshold number of gametocytes (as in Coleman *et al.* 2014). Modifying allocation to transmission is likely to alter transmission rates, but with potentially different consequences in the short- *vs* long-term (Fig. 3). The implications for parasite fitness should be examined by integrating transmission success over the lifespan of infection. For example, curves relating gametocyte abundance to infectivity have already been experimentally-derived (from rodent infections, Bell *et al.* 2012) or statistically-inferred from clinical data (from human infections, Paul *et al.* 2007; Huijben *et al.* 2010). Those curves have been used to estimate the probability of transmission from gametocyte abundance and predict the fitness consequences of different parasite traits (e.g. drug resistance, Huijben *et al.* 2013, developmental synchrony, Greischar *et al.* 2014). The key point is that selection is unlikely to be able to maximize transmission rates and infection length independently, and considering the cumulative transmission success will provide more realistic predictions.

### Intuition: there is a tradeoff between virulence and transmission

This intuition forms the basis for classical theory on virulence evolution (reviewed in Alizon *et al.* 2009), which assumes that within-host proliferation enhances transmission rates but reduces infection length due to host mortality (e.g. Frank 1996; pattern in Fig. 2B). These assumptions hold for some parasites, e.g. HIV, for which higher viral loads correlate with both greater transmission rates and shorter time until the onset of AIDS and death (Fraser *et al.* 2014). Human malaria infections show a more nuanced pattern of host mortality. Severe outcomes are the product of immunity and parasite factors: the likelihood of severe outcomes scales with parasite numbers, but only when parasites exceed a certain threshold biomass (Cunnington *et al.* 2013). Infections are frequently asymptomatic (e.g. Färnert *et al.* 1997; Jafari-Guemouri *et al.* 2006) and terminated by host immunity rather than mortality (e.g. Bruce *et al.* 2000). These asymptomatic hosts can harbour considerable numbers of gametocytes and infect mosquitoes (reviewed in Bousema and Drakeley, 2011). Thus, there is no straightforward association between transmission and the risk of host mortality.

The link between parasite biomass and transmission rate is also less clear in malaria infections, due to the presence of specialized transmission stages. We use a simple model to illustrate how changing transmission investment could alter proliferation in malaria infections (Fig. 3). Constant transmission investment can (with the right parameter values) offset the proliferation of the parasite population so that parasite numbers remain constant (Fig. 3A), and the transient effects of changing transmission investment follow intuition: increasing transmission investment causes a short-term increase in the number of gametocytes (Fig. 3B), while decreasing investment reduces the numbers of gametocytes, at least initially (Fig. 3C). The long-term impact is more complicated, with increased transmission investment actually reducing both proliferative and transmission stage abundance, and decreased transmission investment ultimately resulting in higher parasite biomass and numbers of gametocytes. Rapid initial proliferation – even at the expense of gametocyte production – is predicted to enhance transmission success, since it provides a larger pool of parasites that can subsequently develop into gametocytes (Koella and Antia, 1995). Accordingly, using a quantitative genetics approach we found no evidence of tradeoffs between virulence and transmission in rodent malaria infections, but did uncover tradeoffs between early and late transmission (Mideo *et al.* 2011*a*). When the tradeoff emerges within a trait rather than as a consequence of correlated traits, the optimal transmission rate profile depends on host population dynamics. The size of the host population is predicted to constrain the range of strategies available to parasites (Cressler *et al.* 2015). If hosts are abundant enough, parasites may be able to invade rapidly and persist indefinitely by producing acute infections, from which transmission is highly efficient for only a brief period; when host populations are smaller, parasites producing acute infections are likely to go extinct after depleting the susceptible population, and parasites causing chronic infections should tend to persist (invasion-persistence tradeoff, King *et al.* 2009). Similarly, early transmission is predicted to carry greater benefits when an epidemic is expanding (Day *et al.* 2011; Mideo *et al.* 2011*a*). Since each infection that is ending is replaced by more than one new infection, most infections are in the early stages and the early part of infection has a greater impact on parasite fitness. In contrast, late transmission contributes more to parasite fitness when the infection prevalence attains an endemic equilibrium in the host population (Day *et al.* 2011; Mideo *et al.* 2011*a*). While malaria biology dictates a tradeoff between replication and transmission in the short-term (e.g. Fig. 3), a different pattern may emerge when transmission rates are integrated over the lifespan of an infection. Theory developed to characterize malaria infections supports the idea that common parasite life history traits, including specialized transmission stages, can dissolve the expected tradeoff between virulence and transmission.

### Intuition: within-vector dynamics are unlikely to make a qualitative difference

Many parasites exhibit indirect transmission, so that infecting new hosts requires colonization of one (or more) vectors. To simplify calculations, models commonly use constants to represent transmission to and from the vector, including efforts to estimate disease risk (e.g. Smith *et al.* 2007; Gething *et al.* 2010, 2011) and to characterize the evolutionary consequences of repeated transmission bottlenecks (Chang *et al.* 2013; Chang and Hartl, 2015). Other models instead assume that transmission success scales with total gametocyte production (e.g. to identify optimal transmission investment strategies, Koella and Antia, 1995; Mideo and Day, 2008), but malaria parasites may also defy that logic because not all gametocytes are equally valuable. Their potential contribution to parasite fitness depends on the size and composition of the gametocyte population. Gametocytes develop as male or female and, once they are ingested by a vector, successful fertilization is required for onward transmission (reviewed in Bousema and Drakeley, 2011). Mate finding is likely to be a particular problem when gametocytes (or gametes) are rare, and as a consequence the probability of infecting mosquitoes is observed to accelerate when gametocytes increase from low numbers (e.g. Huijben *et al.* 2010; Bell *et al.* 2012). While greater numbers of gametocytes can improve the odds of transmission, the sex ratio – the proportion of gametocytes that are male – also plays a role. Malaria parasites exhibit female-biased sex ratios (Robert *et al.* 1996; Paul *et al.* 2003; Talman *et al.* 2004), which are predicted to be favourable in inbred populations (local mate competition theory, Hamilton, 1967). When inbreeding occurs it leads to competition for mates between related males which reduces the fitness returns from males relative to females so that female-biased sex ratios will be favoured by selection (Dye and Godfray, 1993; West *et al.* 2000; Nee *et al.* 2002). Female-biased sex ratios occur in infections with low genetic diversity (Read *et al.* 1992, 1995; Paul *et al.* 1995; West *et al.* 2001, 2002; Sowunmi *et al.* 2009), and experimental rodent infections match closely with quantitative theoretical predictions (Reece *et al.* 2008). Yet some malaria parasite species consistently show sex ratios that are far more variable during infection and less female-biased than would be expected from the inbreeding rate (e.g. Shutler and Read, 1998; West *et al.* 2001; Paul *et al.* 2003; Neal and Schall, 2010). The mismatch may be related to the problem of maximizing transmission success when gametocytes are rare. Parasites with a strongly female-biased sex ratio would risk too few males being taken up to fertilize all of the females, a problem that could be exacerbated by transmission-blocking immune defences (such as antibodies that agglutinate male gametes, Carter *et al.* 1979). Accounting for the possibility of too few male gametes when gametocytes are rare, models instead predict sex ratios that are less female-biased than would be predicted from simple theory (Gardner *et al.* 2003, and others), consistent with empirically observed patterns (e.g. Mitri *et al.* 2009; Ramiro *et al.* 2011).

When success is limited by gametocyte numbers, maximizing colonization of the mosquito would seem to be an appropriate target for selection (e.g. Reece *et al.* 2008), but that may not be the case when gametocytes are abundant. Experiments suggest that there is an upper limit on the number of parasites that can successfully develop within the vector (Sinden *et al.* 2007) as well as the number a mosquito can harbour and still survive long enough to transmit (Dawes *et al.* 2009). Because of these limitations, strains that export more gametocytes can actually experience reduced onward transmission (Pollitt *et al.* 2013). A remaining challenge is to determine how often such crowding is likely to constrain parasite fitness, and to what extent parasites can side-step the problem by modifying their gametocyte production, adjusting their sex ratios, or by using apoptosis to correct for over-crowding (reviewed in Pollitt *et al.* 2010; Reece *et al.* 2011). Transmission-blocking interventions will not be effective if parasites are able to compensate for their effects, but even worse would be an intervention that eases crowding within the mosquito and thereby allows more efficient onward transmission. Thus, models and data together suggest that the within-vector dynamics have the potential to make a qualitative difference to parasite evolution, especially in the face of intervention efforts.

## THE ROLE OF MODELS IN DISCERNING THE SELECTIVE LANDSCAPE

Models are uniquely suited to explore the fitness consequences of parasite strategies and to determine whether shifting selection pressures are likely to unearth novel parasite traits. In that vein, models have been used to understand what factors have constrained the evolution of HIV so that vector-transmission is essentially non-existent, despite the potential advantage (Day *et al.* 2008). Similarly, a model of parasites with specialized transmission stages predicted that parasites should divert all resources to transmission investment when infection is ending (terminal investment, Koella and Antia, 1995). Such terminal investment has not been observed (at least, in malaria parasites), even in extremely crowded *in vitro* conditions (Bruce *et al.* 1990). Since the quantitative prediction has not been supported, the model opens up an interesting avenue of questioning. Are there any biases in our experimental or statistical approaches that would obscure terminal investment? Is the predicted strategy not really optimal, and if not, what other factors need to be accounted for in models? If the predicted optimal strategy is correct, what prevents parasites from attaining that evolutionary optimum in reality? By exploring the strategies that have (so far) failed to evolve, models help make sense of the diverse life histories that do exist and the feedbacks that constrain their evolution.

Characterizing the fitness landscape and the constraints on parasite adaptation is a particularly urgent concern, since human interventions are currently remodelling that landscape, easing some constraints and imposing new ones. For example, recent models suggest that sustainable disease control might be achieved by less aggressive vector-control methods (Koella *et al.* 2009; Read and Thomas, 2009). The logic is that malaria parasites, like many other mosquito-borne pathogens, require a long time to incubate within a mosquito in order to be transmitted, so targeting control towards only older mosquitoes (for example through lower doses of insecticides, Glunt *et al.* 2011, or biopesticides with delayed action, Lynch *et al.* 2012) could simultaneously reduce malaria risk while imposing less selection for insecticide resistance in the vector. Thus, mosquitoes would still be present and feeding, but few if any would be capable of transmitting malaria. However, recent experiments suggest that mosquito bites, even from uninfected mosquitoes, can alter within-host dynamics: malaria-infected birds fed upon by uninfected mosquitoes subsequently harboured greater numbers of parasites than birds not receiving mosquito bites, and birds became more infectious in the days following this exposure (Cornet *et al.* 2014). Transient increases in blood-borne parasite abundance are often observed in chronic avian malaria infections and usually attributed to a lapse in host immunity (Valkiunas, 2005), but the experimental results reported by Cornet *et al.* (2014) suggest that such a lapse could be triggered by exposure to mosquito bites. One interpretation of this data is that parasites are able to facultatively alter their dynamics to enhance infectiousness in the presence of mosquitoes, raising the question of how. Do parasites alter transmission investment (e.g. Fig. 3C), or burst sizes (another apparently plastic trait in *P. chabaudi*, Mideo *et al.* 2011*b*), or by switching antigens? Equally important, if any of these strategies could increase infectiousness, why would parasites not always employ them? Cornet *et al.* (2014) use models to show that, if proliferation increases the risk of host mortality, it would select for parasites that only upregulate their numbers when transmission is possible (i.e. when mosquitoes are present), but increasing proliferation late in infection could render parasites more vulnerable to clearance by adaptive immunity, especially if the antigenic repertoire is close to being exhausted.

Distinguishing between these possibilities is essential to predicting the consequences of selectively removing older mosquitoes *vs* conventional approaches aimed at killing all mosquitoes. If parasite expansion is merely the result of removing an immune constraint, then leaving uninfected mosquitoes around could have the unintended consequence of causing infected hosts to relapse, at least in the short-term. Alternatively, if the expansion of parasite numbers represents adaptation on the part of the parasites, killing mosquitoes before they are infectious would remove the benefit of increased proliferation within the host but leave the cost. If increasing parasite numbers late in infection also increases the risk of immune clearance, then less aggressive interventions could be even more efficient at reducing malaria prevalence than the conventional approach of indiscriminately removing all mosquitoes. At the core of the issue is what parasites stand to gain (or lose) from relapses; transient increases in parasite biomass appear advantageous in the short-term, since they coincide with enhanced transmission rates (Cornet *et al.* 2014), but further theory and experiments are needed to project the long-term consequences over the entire parasite life cycle.

## CONCLUSIONS

Models have been immensely useful for refining intuition and improving methods for inferring what cannot be observed directly. Recent work – theoretical and experimental – has highlighted the rich detail of within-host ecology, necessary for placing parasite life cycles into a robust evolutionary framework. These details are likely to matter as disease intervention efforts become more sophisticated, and integration between models and data can locate the critical gaps in knowledge and augment efforts to unravel the complex patterns of parasite evolution.
